# Intraluminal Therapy for *Helicobacter pylori* Infection—Comparison of Medicament Containing Tetracycline, Metronidazole, and Bismuth versus Amoxicillin, Metronidazole, and Clarithromycin: A Randomized Controlled Study

**DOI:** 10.3390/biomedicines11041084

**Published:** 2023-04-03

**Authors:** Ting-Wen Liu, Yen-Po Chen, Cheng-Yu Ho, Ming-Jen Chen, Horng-Yuan Wang, Shou-Chuan Shih, Tai-Cherng Liou

**Affiliations:** 1 Department of Internal Medicine, MacKay Memorial Hospital, Taipei 10449, Taiwan; 2 Department of Medicine, MacKay Medical College, New Taipei City 25245, Taiwan; 3 MacKay Junior College of Medicine, Nursing, and Management, Taipei 112021, Taiwan; 4 Division of Gastroenterology, Department of Internal Medicine, MacKay Memorial Hospital, Tamsui, New Taipei City 25173, Taiwan; 5 Division of Gastroenterology, Department of Internal Medicine, MacKay Memorial Hospital, Taipei 10449, Taiwan

**Keywords:** *Helicobacter pylori*, acidity, transluminal endoscopy, eradication therapy, antibiotics

## Abstract

*Helicobacter pylori (H. pylori)* can be eradicated immediately via local application of single-dose medicament on endoscopic examination. In our previous report, “the eradication rate of intraluminal therapy for *H. pylori* infection (ILTHPI) is 53.7% (51/95) using medicament containing amoxicillin, metronidazole, and clarithromycin”. We aimed to evaluate the efficacy and adverse events of medicament containing tetracycline, metronidazole, and bismuth and to improve the efficacy of stomach acid control before ILTHPI. After usage of dexlansoprazole (60 mg b.i.d.) or vonoprazan (20 mg q.d.) for 3 days before ILTHPI, 103 of 104 (99.1%) symptomatic *H. pylori*-infected treatment-naïve patients achieved levels of stomach pH ≥ 6. Patients were randomized to receive ILTHPI with medicaments containing tetracycline, metronidazole, and bismuth (Group A, *n* = 52) or amoxicillin, metronidazole, and clarithromycin (Group B, *n* = 52). The eradication rate of ILTHPI was similar between Group A (76.5%; 39/51) and Group B (84.6%, 44/52) (*p* = 0.427) and the adverse event was mild diarrhea (2.9%; 3/104). The eradication rate significantly increased from 53.7% (51/95) to 84.6% (44/52) after acid control (*p* = 0.0004) for Group B patients. The overall eradication rates of successful ILTHPI plus 7-day non-bismuth (Group A) or 7-day bismuth (Group B) oral quadruple therapy for ILTHPI failure patients were both excellent (96.1% for Group A and 98.1% for Group B).

## 1. Introduction

“*Helicobacter pylori* (*H. pylori*) is the most common chronic bacterial infection in humans, about half of the global population is infected with *H. pylori*” [1]. “*H. pylori* is an important causal factor of chronic gastritis, peptic ulcer disease, gastric cancer, and gastric mucosa-associated lymphoid tissue lymphoma [2,3]. The eradication of *H. pylori* can relieve the peptic ulcer disease and decrease the risk of gastric cancer” [3,4]. “*H. pylori* reside in the acid and mucous layer of the human gastric mucosa, adheres to and colonizes the mucosal surface of the stomach” [5]. Due to the special gastric milieu, no single-dose oral agent can eradicate *H. pylori* immediately. However, the prolonged usage of multiple-dose antibiotics leads to the global emergence of *H. pylori* antibiotic resistance [6,7,8]. In a recent meta-analysis of the resistance patterns of *H. pylori* strains in the United States between 2011 and 2021, the pooled rates of resistance were 42.1% for metronidazole, 37.6% for levofloxacin, and 31.5% for clarithromycin [7]. In Taiwan, progressively higher primary resistance rates were also observed; the rates of resistance were 42.3% for metronidazole, 38.8% for levofloxacin, and 20.4% for clarithromycin in 2019 [8]. Several oral antibiotic regimens are no longer appropriate as the first-line treatment due to the <80% eradication rate in most areas [3,9,10]. The WHO has listed *H. Pylori* as one of 12 antibiotic-resistant bacteria that have the greatest threat to human health in February 2017, which calls for alternative novel therapy [11].

We previously demonstrated that “*H. pylori* can be eradicated immediately via single-dose medicaments applied while conducting an endoscopic examination. After intraluminal therapy for *H. pylori* infection (ILTHPI) with medicament containing amoxicillin, clarithromycin, and metronidazole, the immediate eradication rate is 53.7% (51/95) [12], and significantly higher (72%; 36/50) for patients with gastric juice pH at or above 4” [13]. To achieve an appropriate eradication rate (≥80%), a control of stomach pH at or above 4 for patients prior to ILTHPI is strongly recommended. However, due to the steadily rising global antibiotic resistance to clarithromycin and levofloxacin, current guidelines recommend bismuth quadruple therapy as first-line empiric therapy in most areas [3,9,10]. Accordingly, there is also a pressing need to develop other medicaments for the ILTHPI. The primary aim of the current study was to evaluate and compare the efficacy and adverse events of medicament containing tetracycline, metronidazole, and bismuth versus amoxicillin, metronidazole, and clarithromycin for the ILTHPI. The secondary aim was to control the stomach pH at or above 4 with a proton pump inhibitor (PPI) or potassium-channel acid blocker (P-CAB) for 3 days prior to the ILTHPI.

## 2. Materials and Methods

### 2.1. Patients

**Figure 1:**
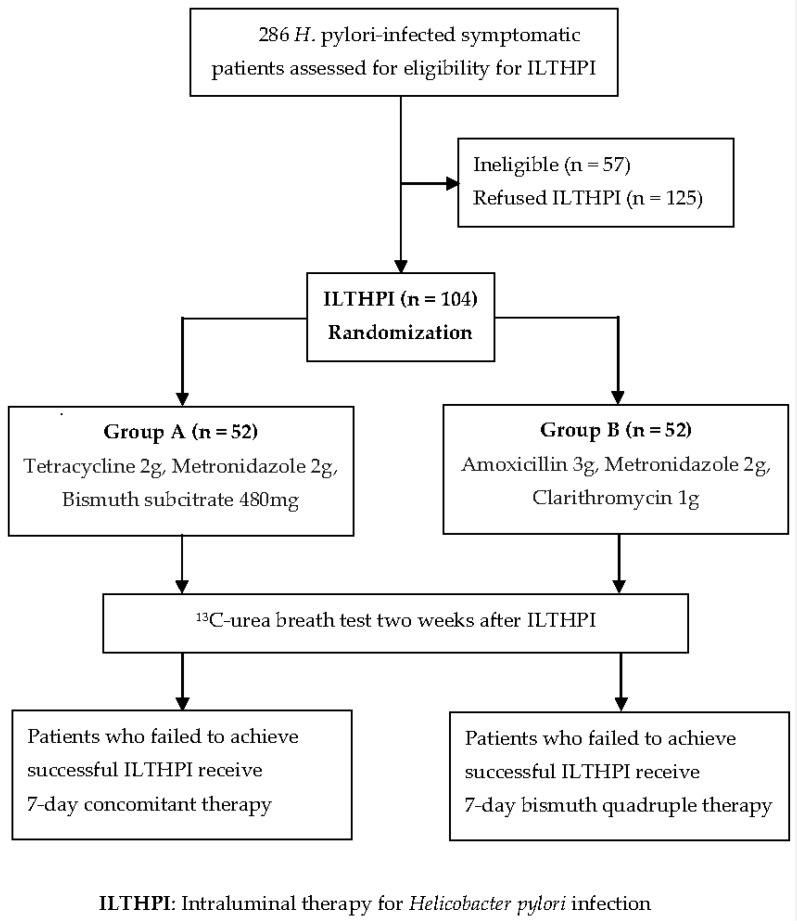
Study flow chart.

This clinical trial is a single-center prospective randomized controlled study (NCT04853875). From April 2021 to July 2022, 286 consecutive *H. pylori*-infected patients with upper abdominal pain or discomfort were assessed for eligibility for ILTHPI, and those eligible were invited to receive ILTHPI before oral antibiotic therapies. All the 286 patients, aged between 20 and 75, were positive for ^13^C-UBT. Patients with any of the following were excluded from the study, with the same criteria as our previous studies [12,14]. “(1) contraindication of endoscopic examination; (2) previous gastric surgery; (3) gastroduodenal deformity, stenosis, or obstruction; (4) gastroduodenal malignancy; (5) previous eradication therapy for *H. pylori*; (6) use of antibiotics or bismuth salts in the previous 4 weeks; (7) use of PPI or H_2_-blocker in the previous 2 weeks; (8) previous allergic history to medications used for the ILTHPI; (9) pregnant or lactating women; (10) severe concurrent diseases (such as advanced renal disease or decompensated liver cirrhosis) or malignancy; and (11) inability to give written informed consent or refused ILTHPI”. We excluded 125 patients who refused ILTHPI and 57 ineligible patients, including “(1) 8 contraindication of endoscopic examination; (2) 3 previous gastric surgery; (3) 2 gastroduodenal deformity, stenosis, or obstruction; (4) 2 gastroduodenal malignancy; (5) 11 previous eradication therapy for *H. pylori*; (6) 4 use of antibiotics or bismuth salts in the previous 4 weeks; (7) 18 use of PPI or H_2_-blocker in the previous 2 weeks; (8) 2 previous allergic history to medications used; (9) 1 lactating woman; (10) 5 severe concurrent diseases (2 CKD stage 4, 1 CKD stage 5, 1 decompensated liver cirrhosis, 1 malignancy); and (11) 1 inability to give written informed consent”. As shown in Figure 1, we enrolled a total of 104 *H. pylori*-infected treatment-naïve patients for ILTHPI. All participants provided written informed consent before enrollment, and the study was approved by the Institutional Review Board of our hospital (IRB Number: 21MMHIS016e).

### 2.2. Methods

We used the permuted block randomization (block number = 4) available from https://www.sealedenvelope.com/simple-randomizer/v1/lists [accessed on 18 April 2021; se_list_233653209030622] to conduct the open-labeled randomization. Patients who enrolled for the ILTHPI were open-label randomized into Group A and Group B. The 52 patients in Group A received ILTHPI with medicament containing 2 g of tetracycline powder in capsules, 2 g of metronidazole powder in capsules, and 480 mg of crushed bismuth subcitrate film-coated tablets in powder form. The other 52 patients in Group B received ILTHPI with medicament containing 3 g of amoxicillin powder in capsules, 2 g of metronidazole powder in capsules, and 1 g of crushed film-coated clarithromycin tablets in powder form. We tried to control the gastric juice pH at or above 4 for 3 days before the ILTHPI. Patients in Group A or Group B were further randomized to receive either dexlansoprazole (60 mg delayed release capsule) one capsule twice a day or vonoprazan (20 mg film-coated tablet) one tablet per day. According to our previous study designs [12,14], “the clinical characteristics, endoscopic findings, and levels of gastric juice pH were recorded. All the procedures of ILTHPI were performed by a single, experienced senior gastroenterologist, and were strictly followed as detailed in our previous report [12]. For all participants, a high-resolution electronic endoscope from Olympus Co. (Tokyo, Japan, GIF 260 or 290 series) was used to detect and interpret mucosal changes, such as ulceration or hyperemia. Levels of gastric juice pH were detected, and the methodology was detailed in our previous report [13]. The pH of gastric juice was measured using two pH strips (Macherey-Nagel pH-Fix 0.0–6.0 and pH-Fix 4.5–10.0); each scaled in pH 0.5 intervals. Levels of gastric juice pH in each patient are classified into three ranges, including normal acidity (pH < 4.0), low-level hypoacidity (pH 4.0 to 5.5), and high-level hypoacidity (pH ≥ 6.0)”. 

The procedures of ILTHPI and the post-ILTHPI follow-ups of the patients were strictly followed as our previous designs [12,14]. “Patients took two orally disintegrating lansoprazole tablets (30 mg each) before ILTHPI and 8 to 10 h after ILTHPI”. “During ILTHPI, an endoscopic apparatus and washing pipe from Olympus Co. (Tokyo, Japan, EndoTherapy product name: PW-1L-1) were used to irrigate the gastric mucus with acetylcysteine (12 mg/mL) solution to remove the mucus on the gastric mucosa. The total dosage of acetylcysteine was less than 140 mg/kg”. “The antibiotics powder for the ILTHPI was mixed with 80 mL (8 g) of sucralfate suspension and 200 mL of distilled water and was applied to all surfaces of the gastric mucosa and the duodenal mucosa of the duodenal bulb as evenly as possible using the same washing pipe”. “During the endoscopic examination and ILTHPI, each patient was sedated with intravenous midazolam (5 mg) and fentanyl citrate (0.05 mg or 0.1 mg). Vital signs were closely monitored using a physiological monitor, and the safety profiles were strictly followed as defined in our previous report” [12]. “After ILTHPI, patients were asked to rest for 30 min for the effects of sedation to wear off before leaving. However, they were allowed to take meals if they did not experience abdominal discomfort. The post-ILTHPI follow-ups were performed by other physicians. Patients were scheduled for consultation 3 to 7 days after ILTHPI and all patients were asked to report presence or absence of adverse events during the consultation”. “The ^13^C-UBT was used to assess the successful eradication of *H. pylori* 2 weeks after ILTHPI. The eradication rate of ILTHPI was calculated for per-protocol patients, and those who failed to return for follow-up ^13^C-UBT were excluded. All patients with successful ILTHPI underwent subsequent stool *H. pylori* antigen tests (Artron *H. pylori* stool Ag rapid test; Artron Laboratory Inc., Canada) 4 to 6 months after ILTHPI to exclude short-term recurrence of *H. pylori* infection”. 

“Patients who failed to achieve immediate *H. pylori* eradication after ILTHPI received 7-day concomitant therapy (lansoprazole 30 mg b.i.d., amoxicillin 1000 mg b.i.d., metronidazole 500 mg b.i.d., and clarithromycin 500 mg b.i.d.) for Group A, or 7-day bismuth quadruple therapy (lansoprazole 30 mg b.i.d., tetracycline 500 mg q.i.d., metronidazole 500 mg t.i.d. and bismuth subcitrate 120 mg q.i.d.) for Group B. Patient compliance and adverse events of oral antibiotic therapies were evaluated, and the *H. pylori* status was assessed by the ^13^C-UBT 4 weeks after the completion of treatment”. For ethical considerations and also under the impact of the COVID pandemic in our country, we did not conduct randomized oral antibiotics therapy (7-day concomitant versus 7-day bismuth quadruple therapy) for the 125 patients who declined ILTHPI.

### 2.3. Statistical Analysis 

According to our previous report, “the eradication rate of ILTHPI with medicaments containing antibiotic powders of amoxicillin (3 g), metronidazole (2 g), and clarithromycin (1 g) for patients without stomach pH control is 53.7% (51/95)” [12]. We expected that the eradication rate of Group B patients was appropriate (≥80%) after controlling the stomach pH for 3 days prior to the ILTHPI. We used the Sample Size Calculator (Kane SP. Sample Size Calculator. ClinCalc: available from https://clincalc.com/stats/samplesize.aspx [accessed on 11 January 2021]) to determine the minimum number of 52 subjects enrolled in Group B in order to have sufficient statistical power to detect a difference (α = 0.05, power = 0.8).

Methods of statistics were also similar to our previous report [12]. “Unless otherwise indicated, values were expressed as mean ± standard deviation (SD). Both intention-to-treat (ITT) and per-protocol (PP) analyses were conducted for patients who received oral antibiotic therapy. The ITT analysis included all patients who received therapies; and those who failed to do follow-up therapy or tests were considered treatment failures. The PP analysis excluded patients who failed to do follow-up therapy or tests as per protocol. Categorical data were compared using the χ^2^ test or Fisher’s exact test as appropriate. Continuous variables were expressed as mean ± standard deviation. Student’s t-test was used to compare the mean values of continuous variables. Corresponding 95% confidence intervals (95% CI) were calculated for all estimates. All reported *p*-values were based on two-sided tests and considered statistically significant if less than 0.05. Data were analyzed by using SPSS version 22 (SPSS Inc., Chicago, IL, USA)”.

## 3. Results

All 104 enrolled patients completed the ILTHPI with good safety profiles and without violation of any of the safety indicators. The average duration of ILTHPI was 8 min and 32 s, including the average time spent for the irrigation of gastric mucus (4 min and 38 s) and for the application of medicaments (3 min and 52 s). Table 1 showed the clinical characteristics of patients in Group A and Group B. There are no significant differences (*p* > 0.05) in any of the demographic characteristics between patients in Group A and Group B including age, gender, smoking, users of alcohol, tea, coffee, non-steroid anti-inflammatory drugs (NSAID), and the body mass index (BMI).

All patients in Group A achieved gastric juice pH levels at or above 6, including the usage of dexlansoprazole (26 patients) or vonoprazan (26 patients) prior to the ILTHPI. One patient in Group B was detected to have gastric juice pH at a level of 1.5 even after the usage of vonoprazan prior to the ILTHPI. The remaining 51 patients achieved gastric juice pH levels at or above 6, including the usage of dexlansoprazole (26 patients) or vonoprazan (25 patients). 

Table 2 revealed there are no significant differences (*p* = 0.946) in endoscopic findings regarding proportions of peptic ulcer disease, gastritis, or normal appearance between Group A and Group B. Sub-classification of endoscopic gastritis also revealed no significant differences (*p* = 0.914) between the two groups regarding the distributions of endoscopic features of gastritis (hyperemic mucosal change) in the antrum, corpus, and cardia.

As shown in Table 3, one patient in Group A dropped out without subsequent ^13^C-UBT, and the other 103 patients underwent subsequent ^13^C-UBT between two to four weeks after ILTHPI. A total of 39 of 51 patients (76.5%; 95% CI: 63.1% to 86.1%) in Group A and 44 of 52 patients (84.6%; 95% CI: 72.2% to 92.3%) in Group B achieved successful ILTHPI. There is no significant difference in the eradication rate between Group A and Group B (*p* = 0.427). All patients with successful ILTHPI underwent subsequent stool *H. pylori* antigen tests 4 to 6 months after ILTHPI, and no short-term recurrence of *H. pylori* infection was observed. One patient in Group A (1.9%; 95% CI: <0.01% to 11.1%) and two patients in Group B (3.8%; 95% CI: 0.3% to 13.7%) had watery diarrhea 3–5 times in 1 or 2 days following the ILTHPI; there is no significant difference for the incidence rate of the adverse event between Group A and Group B (*p* = 1.0). The overall incidence rate of adverse events was 2.9% (3/104; 95% CI: 0.6% to 8.5%) and the severity of adverse events after ILTHPI was mild as those of our previous reports [12,14]. We must mention that the potential side effects of sedated endoscopy (nausea and dizziness) were overcome in this study after we provided a more comfortable recovery room (quiet and dark place) and a longer wait time for the sedation to wear off.

Table 4 revealed the overall eradication rate of ILTHPI plus oral antibiotic therapy for patients who failed ILTHPI. All 20 patients completed the rescue oral antibiotic therapies with good compliance (took ≥ 80% of drugs) and no adverse event was observed. All patients completed subsequent ^13^C-UBT. The eradication rates, either for ITT or PP, were 83.3% (10/12; 95% CI: 54.0% to 96.5%) for 7-day concomitant therapy (Group A) and 87.5% (7/8; 95% CI: 50.8% to 99.9%) for 7-day bismuth quadruple therapy (Group B). There is no significant difference in the eradication rate of oral antibiotic therapy comparing Group A versus Group B (*p* = 1.0). The overall eradication rate of ILTHPI plus oral antibiotic therapy is 96.1% (49/51; 95% CI: 86.0% to 99.7%) for Group A and 98.1% (51/52; 95% CI: 88.9% to >99.9%) for Group B. There is no significant difference in the overall eradication rate between Group A and Group B (*p* = 0.618).

Table 5 showed the clinical characteristics, the percentage of high-level hypoacidity (pH ≥ 6.0), and the eradication rates of ILTHPI using medicaments containing powders of amoxicillin (3 g), metronidazole (2 g), and clarithromycin (1 g) of the 100 patients (Group A) without stomach acid control in our previous reports [12,13]. The percentage of high-level hypoacidity (Gastric Juice pH ≥ 6.0) was 45.0% (45/100; 95% CI: 35.6% to 54.8%). Five patients were lost to follow-up, the eradication rate was 53.7% (51/95; 95% CI: 43.7% to 63.4%). As compared to the 52 patients (Group B) with stomach acid control in the current study, there are no significant differences in all clinical characteristics between Group A and Group B (*p* > 0.05). However, there is a significant difference in the percentage of high-level hypoacidity (45.0% [45/100] vs. 98.1% [51/52]; *p* < 0.0001) comparing Group A versus Group B. The eradication rate is also significantly higher for Group B patients than Group A (84.6% [44/52] vs. 53.7% [51/95]; *p* = 0.0004, α = 0.05, power = 0.98).

## 4. Discussion

The indications for *H. pylori* eradication and endoscopic examination are variant in the different geographic areas owing to the differences in the prevalence of *H. pylori* infection and gastric cancer. Meanwhile, the availability of medications, the cost of endoscopic examinations, and the facilities of health insurance systems are also important factors. It is widely accepted that endoscopy is recommended for dyspeptic patients older than 50 (45–55) years [10], particularly with coexisting “alarm symptoms (bleeding, anemia, unexplained weight loss, odynophagia, progressive dysphagia, persistent vomiting, and family history of gastric cancer)” [3,10]. In addition, patients receiving endoscopic examinations to detect early gastric cancer are increasing due to the aging of the population, the economic improvement of countries, and the establishment of National Health Insurance (NHI). In Taiwan, “the NHI system provides medicaments for *H. pylori* eradication and endoscopic examinations for pretreatment screening and post-treatment surveillance due to the high prevalence of *H. pylori* infection and gastric cancer in Taiwan” [9,15].

Current global guidelines for the indications of *H. pylori* eradication are gradually expanding [3,9,10]. “However, there has so far been no definitive evidence of immediate eradication of *H. pylori* from single-dose oral therapeutic agents due to the special gastric milieu of *H. pylori*” [13]. Most oral antibiotics are enteric-coated or encapsulated due to their instability and degradation under normal gastric pH. Moreover, “approximately 80% of *H. pylori* reside in the mucus layer of the human stomach which is difficult for the penetration of antibiotics” [13]. It is impossible to eradicate *H. pylori* in a short period of time via antibiotic absorption through the intestinal tract and redistribution to the stomach lumen due to insufficient drug concentration in the gastric mucus layer and acid layer. In view of the heavy expenses *H. pylori* eradication incurs in health systems, the necessity of multiple-dose oral antibiotics therapies, the inconvenience and adverse events of oral therapies, and the steadily going global antibiotic resistance, we developed the ILTHPI. The procedures of ILTHPI include “the control of intragastric pH within a certain range, irrigation of the gastric mucosal surface with a mucolytic agent, and direct application of a single-dose medicament containing antimicrobial agents” [12]. The patient can achieve concomitant eradication of *H. pylori* with single-dose medicament on endoscopic examination, which eliminates the necessity of subsequent multiple-dose oral antibiotics therapies and reduces the burdens of treatment for individuals and global NHI systems. Compared to the systemic therapy with multiple-dose oral antibiotics, “the ILTHPI provides a single-dose local therapy through the administration of a single-dose medicament directly to the mucosal surface colonized by the *H. pylori*, issues commonly associated with the use of multiple-dose oral antibiotics regarding drug absorption, tissue redistribution, liver and kidney injuries, systemic side effects, and patient compliance could be reduced” [12,14]. Moreover, “there are no significant influences of the other factors including age, gender, BMI, type of disease (type 2 DM, dyslipidemia, renal function, anemia), personal habits (smokers, users of alcohol, tea, coffee, NSAID, steroid, statin), and endoscopic findings (gastric ulcer and/or duodenal ulcer, inflammation of cardia)” [14]. In addition, “all patients completed the procedures of ILTHPI with good safety profiles and only mild adverse events. The medicaments for the ILTHPI in our study were without significant risk of kidney and liver injuries [12]. ILTHPI may have the function to help decrease the bacterial load to a level that can shorten the duration of subsequent rescue oral antibiotics therapies to one week [12] and may also have the potential to diminish the increasing rate of global antibiotic resistance” [14]. 

The limitations in the current research were similar to our previous studies. “First, to evaluate the impact of gastric juice pH and as a first-line therapy, we only enrolled *H. pylori*-infected treatment-naïve patients and excluded some patients who are actually suitable for the procedures of ILTHPI, including the 11 previous eradication therapy for *H. pylori* and the 18 use of PPI or H2-blocker in the previous 2 weeks. Second, we did not enroll patients who declined ILTHPI to receive randomized oral antibiotics therapies (concomitant or bismuth quadruple therapy) due to the influence of the COVID pandemic in our country. Third, from an ethical standpoint, given that the efficacy of medicament containing tetracycline, metronidazole, and bismuth was unknown, we only enrolled a limited number of 52 patients in each group. Furthermore, antibiotic resistance is a crucial factor affecting the eradication rate, however, the antibiotic susceptibility tests (AST) were still not available in our medical center, our NHI systems, and most medical institutions due to the possibility of false negatives and problems of clinical applicability including the cost” [16]. As mentioned in our previous studies, “The crater of peptic ulcer and the inflammation of cardia might have a negative impact on the success of ILTHPI due to technique difficult locations for the procedures of ILTHPI including the irrigation of mucus and the application of medications” [12,14]. In addition, hyperemic change of gastric cardia indicates a higher bacterial load in the cardia, leading to failure of the ILTHPI. Due to the limited number in our study, we classified “the endoscopic findings of *H. pylori* infection regarding the presence of peptic ulcer disease, gastritis, or normal appearance and sub-classification the distributions of endoscopic features of gastritis (hyperemic mucosal change) in antrum, corpus, and cardia” [12,14]. However, other classifications of endoscopic findings remained to be studied if more patients had enrolled, including the presence of sticky mucus, diffuse redness, spotty redness, mucosal swelling, chicken skin-like nodularity, enlarged folds, mucosal atrophy, and intestinal metaplasia.

Many studies have suggested that “maintenance of intragastric pH at ideally 4 or above stabilizes the pharmacological properties of the administered antibiotics [17,18,19], and that sustained control of intragastric pH at 6 or above stimulates the replication of *H. pylori* and increases the bactericidal efficacy of oral antibiotics” [17,19,20]. Our previous studies also revealed that “the eradication rate of ILTHPI with medicament containing amoxicillin, metronidazole, and clarithromycin is significantly higher in patients with gastric juice pH at or above 4 (72% [36/50] vs. 33.3% [15/45]; *p* < 0.001)” [13]. To achieve an appropriate (≥80%) eradication rate of ILTHPI, a more effective strategy to control the stomach pH at or above 4 prior to ILTHPI is strongly recommended. In this study, we tried to control the gastric juice pH at or above 4 using dexlansoprazole (60 mg b.i.d.) or vonoprazan (20 mg q.d.) for 3 days before the ILTHPI. Almost all patients (99.1% [103/104] 95% CI: 94.2% to >99.9%) achieved levels of stomach pH at or above 6, including the usage of dexlansoprazole (100% [52/52]) or vonoprazan (98.1% [51/52]). Although one patient in Group B was detected to have gastric juice pH at a level of 1.5 even after the usage of vonoprazan prior to the ILTHPI, she still achieved successful ILTHPI. As compared to our previous report [12], after the usage of dexlansoprazole or vonoprazan for 3 days prior to the ILTHPI, the eradication rate significantly increased from 53.7% (51/95) to 84.6% (44/52) (*p* = 0.0004) (Table 5). Although vonoprazan (a potassium-competitive acid blocker) can achieve more rapid and sustained control of stomach pH and is not significantly affected by CYP2C19 polymorphisms [21], it is currently not available in many areas. The usage of dexlansoprazole (60 mg twice daily) for 3 days prior to the ILTHPI may be a second choice. Further studies for the efficacy of acid control with vonoprazan or dexlansoprazole twice daily for one to two days may shorten the duration of acid control prior to the ILTHPI. In addition, the impact of stomach pH on the medicament containing tetracycline, metronidazole, and bismuth remained to be studied.

The first-line treatment of *H. pylori* infection remains empiric in most areas despite recent advances in antibiotic susceptibility testing. “Due to the global increasing antibiotic resistance rates for clarithromycin, metronidazole, and levofloxacin [6,7,8], several national and international guidelines have suggested a 14-day bismuth quadruple therapy containing tetracycline, metronidazole, and bismuth as first-line therapy for the empiric treatment of *H. pylori* infection” [3,9,10]. Our previous study revealed that “*H. pylori* can be eradicated with mono-antibiotic ILTHPI, the efficacy of each mono-antibiotic ILTHPI was 20% (4/20) for metronidazole, 10% (2/20) for amoxicillin, and 5% (1/20) for clarithromycin. The eradication rate of metronidazole is higher than amoxicillin and clarithromycin” [14]. The in vitro metronidazole resistance can be overcome due to the extremely high local concentration of antibiotic applied by the ILTHPI. “Metronidazole is relatively stable in an acidic environment (pH 2.0–7.0) and with ~800-h degradation half-life in gastric juice samples at a pH level of 2.0” [18]. Moreover, “metronidazole has a molecular weight of 171.15 g/mol, which is relatively a small particle size compared with other medicaments (365.4 g/mol of amoxicillin and 748 g/mol of clarithromycin) [22,23,24]. The small size allows metronidazole to act by being taken up by organisms through passive diffusion and was then reduced by electron-transport proteins to an active intermediate product. The reduced metronidazole is cytotoxic which causes DNA strand breaks, thus inhibiting DNA synthesis and cell growth” [24]. The above-mentioned viewpoints indicated that metronidazole is suitable for ILTHPI. 

In addition, “the eradication rate of ILTHPI is significantly higher in patients with triple-antibiotic ILTHPI than mono-antibiotic ILTHPI (53.7% vs. 11.7%; *p* < 0.0001; α = 0.05, power = 1.0)”, which suggested that metronidazole-containing triple antibiotic and/or antimicrobial agents should be effective for the ILTHPI [14]. Given the low resistance rate of tetracycline [6,7,8] and the reliably higher eradication rate of bismuth quadruple therapy [25,26,27], the efficacy of medicament containing metronidazole, tetracycline, and bismuth for the ILTHPI should thus be evaluated. 

As shown in Table 3, our study is the first report showing that ILTHPI with medicament containing tetracycline, metronidazole, and bismuth is effective and achieves a 76.5% eradication rate after control of the stomach pH at or above 6. There is no significant difference as compared with the medicament containing amoxicillin, metronidazole, and clarithromycin (76.5% vs. 84.6%; *p* = 0.427). Moreover, the adverse events of both regimens are equally minimal (1.92%; vs. 3.85%; *p* = 1.0). For *H. pylori*-infected patients, when the indications of endoscopy come, the concomitant ILTHPI using both medicaments can provide a 76.5% or 84.6% opportunity to eradicate *H. pylori* immediately and eliminate the necessity of subsequent multiple-dose oral antibiotic therapy, which is of grave importance in view of the heavy expenses *H. pylori* eradication incurs in health systems and the increasing global antibiotic resistance due to prolonged oral antibiotic therapies. 

Current guidelines [3,9,10] recommend “a 14-day bismuth quadruple therapy for the first-line treatment in areas of high (>15%) or unknown clarithromycin resistance. If this is not available, a 14-day concomitant therapy may be considered” [10]. However, a 14-day duration of treatment may lead to issues of patient compliance and adverse events, which negatively impacts the eradication rate. Our previous study indicated that “ILTHPI may have the function to help decrease the bacterial load to a level that can shorten the duration of subsequent rescue oral antibiotics therapies from 14-day to 7-day” [12]. In this study, we designed 7-day concomitant and 7-day bismuth quadruple therapies, respectively, for Group A and Group B patients who failed ILTHPI. As shown in Table 4, the eradication rate of rescue oral therapy is equally appropriate (>80%) for Group A and Group B (83.3% vs. 87.5%; *p* = 1.0); the compliance was good, and no adverse events were observed in both groups due to the shortening of the therapeutic duration from 14 days to 7 days. In addition, the overall eradication rates of both therapeutic sequences are optimal (>90%). There is no significant difference comparing Group A versus Group B (96.1% vs. 98.1%; *p* = 0.618). Based on these results, we recommend ILTHPI with medicament containing tetracycline, metronidazole, and bismuth for patients in geographical areas of high (>15%) or unknown clarithromycin resistance, patients who have had previous exposure to clarithromycin, or who are allergic to penicillin. However, we excluded patients allergic to penicillin in this study, as the optimal rescue regimens for such patients remain to be studied. 

Our previous studies showed that “a single-dose antibiotic applied in the ILTHPI may not induce secondary antibiotic resistance as compared to the multiple-dose oral antibiotic therapies [14], and can shorten the duration of subsequent rescue oral antibiotics therapies from 14-day to 7-day” [12]. As a local therapy, ILTHPI may have the potential to diminish the increasing rate of global antibiotic resistance. Although antibiotic resistance should be considered a crucial factor affecting the eradication rate of ILTHPI, a recent meta-analysis displayed that “Susceptibility-guided treatment is not better than empirical treatment of *H. pylori* infection in first-line therapy if the most updated quadruple regiments are empirically prescribed” [16]. We used the most updated quadruple regimens for the therapeutic sequences in treatment-naïve patients; the antibiotic susceptibility tests (AST) may not be necessary for the current study. “However, due to the global increasing primary resistance rate for clarithromycin, metronidazole, and levofloxacin, to achieve a higher eradication rate of ILTHPI, selection of optimal antibiotics in accordance with the geographical surveillance of antibiotic resistances or based on pre-ILTHPI molecular stool AST [28,29,30] is extremely important”. Meanwhile, the gastric juice obtained during the ILTHPI could also provide susceptibility-guided therapy for patients who failed ILTHPI [31,32]. 

Current antibiotics suitable for *H. pylori* eradication are limited (clarithromycin, metronidazole, levofloxacin, amoxicillin, tetracycline, and rifabutin). Since ILTHPI is a local therapy, the efficacy of antibiotics and antimicrobial agents are quite different between the ILTHPI and traditional oral antibiotic therapies due to the different routes of administration. The possible effective agents for ILTHPI could be any kind of penicillin, cephalosporin, macrolide, tetracycline, lincosamide, nitroimidazole, quinolone, rifabutin, furazolidone, bismuth compounds, and, especially, in vitro susceptibility testing is effective for *H. pylori.*

Since “*H. pylori* has a doubling time of 4–6 h [33], stimulating the replication of *H. pylori* with high dose PPI or potassium-competitive acid blockers (p-CABs) and sustained retention of applied medicaments in the stomach lumen for at least 4–6 h or at best 12–18 h are required to reach its best efficacy in the ILTHPI” [12,13,14]. Further investigations are necessary regarding the development of more specified intraluminal antibiotics/antimicrobial medicaments without or with carriers/polymers (such as liquid, emulsion, suspension, or gel formula, dual-release medicaments, antibiotic-loaded polymers, antibiotic-loaded biodegradable microspheres, or antibiotic-loaded nanoparticles), other combined antibiotics and/or antimicrobial complex with a synergistic effect, and the supplementation of suitable probiotics. The above-mentioned antibiotics and anti-microbial agents may cross-link to or be mixed with polymers or carriers conferring high affinity to the gastric mucosal surface. 

Moreover, the usage of other mucolytic agents (such as ambroxol, carbocisteine, erdosteine, mecysteine, or dornase alfa) for patients with sticky mucus, the invention of suitable devices specifically tailored to the ILTHPI (such as a more effective pumping machine, a more powerful shower nozzle for irrigation, and a specifically designed sprayer nozzle for the application of medicaments) should be beneficial to shorten the duration of ILTHPI and improve its eradication rate. 

## 5. Conclusions

In conclusion, the usage of dexlansoprazole (60 mg b.i.d.) and vonoprazan (20 mg q.d.) for 3 days prior to the ILTHPI is extremely effective to control the gastric juice pH at or above 6 (99.1%; 103/104), and significantly increased the eradication rate of medicament containing amoxicillin, metronidazole, and clarithromycin (53.7% vs. 84.6%; *p* = 0.0004). ILTHPI with medicament containing tetracycline, metronidazole, and bismuth is effective (76.5%; 39/51) and the adverse event is minimal (1.9%; 1/52). Using bismuth and non-bismuth quadruple regimens for the therapeutic sequence of ILTHPI and a 7-day rescue oral antibiotics therapy achieved a superior (96.1%) overall eradication rate, and a reverse therapeutic sequence also achieved an excellent (98.1%) overall eradication rate (Table 6).

## Figures and Tables

**Table 1:** Clinical characteristics of *Helicobacter pylori* infected patients.

Characteristics	Group A (*n* = 52)	Group B (*n* = 52)
Age (years, mean ± SD/range) *	49.73 ± 12.96 (20–73)	50.58 ± 11.37 (25–72)
Gender (M/F) *	30/22	30/22
NSAID ingestion *	11 (21.2%)	12 (23.1%)
Smoking *	8 (15.4%)	10 (19.2%)
Alcohol consumption *	7 (13.5%)	6 (11.5%)
Ingestion of tea *	18 (34.6%)	19 (36.5%)
Ingestion of coffee *	22 (42.3%)	20 (38.5%)
BMI (kg/m^2^, mean ± SD/range) *	25.4 ± 4.5 (17.5–37.2)	25.7 ± 4.8 (17.8–38.5)

SD: standard deviation. NSAID: non-steroid anti-inflammatory drug. BMI: Body Mass Index. * *p* > 0.05 for all clinical characteristics between Group A and Group B.

**Table 2:** Endoscopic findings of *Helicobacter pylori* infected patients.

Endoscopic Findings	Group A (*n* =52)	Group B (*n* = 52)
Normal *	7 (13.5%)	6 (11.5%)
Gastritis *	45 (86.5%)	46 (88.5%)
(antrum **/corpus **/cardia **)	(40/44/32)	(38/45/35)
Peptic ulcer disease *	14 (26.9%)	15 (28.8%)

* *p* > 0.05 for proportions of peptic ulcer disease, gastritis, or normal appearance between Group A and Group B. ** *p* > 0.05 for the distribution of endoscopic findings of gastritis in antrum, corpus, and cardia between Group A and Group B.

**Table 3:** The eradication rate and adverse event of ILTHPI.

Medicaments	Patients Number (Lost to Follow up)	Eradication Rate	Adverse Event
**Group A ^†^**	52 (1)	39/51 (76.5%) *	1/52 (1.9%) **
**Group B ^†^**	52 (0)	44/52 (84.6%) *	2/52 (3.8%) **

ILTHPI: intraluminal therapy for *Helicobacter pylori* infection. ^†^ Group A: Medicaments containing tetracycline, metronidazole, and bismuth. ^†^ Group B: Medicaments containing amoxicillin, metronidazole, and clarithromycin. * *p* = 0.427 for the eradication rate comparing Group A versus Group B. ** *p* = 1.0 for the adverse event comparing Group A versus Group B.

**Table 4:** The overall eradication rate of intraluminal therapy plus oral antibiotic therapy.

Patients (No.)	ILTHPI *	Oral Antibiotic Therapy ^†^*	ILTHPI Plus Oral Antibiotic Therapy (Overall Eradication Rate) *
Group A (51)	39/51 (76.5%)	10/12 (83.3%)	49/51 (96.1%)
Group B (52)	44/52 (84.6%)	7/8 (87.5%)	51/52 (98.1%)

ILTHPI: Intraluminal therapy for *Helicobacter pylori* infection. ^†^ Oral antibiotic therapy for patients failed ILTHPI (Group A: 7-day concomitant therapy, Group B: 7-day bismuth quadruple therapy). * *p* > 0.05 for the eradication rate of ILTHPI, oral antibiotic therapy, or ILTHPI plus oral antibiotic therapy between Group A and Group B.

**Table 5:** Eradication rates of ILTHPI without and with stomach acid control.

Clinical Characteristics	Group A ^†^ (*n* = 100)	Group B ^†^ (*n* = 52)
Age (years, mean ± SD/range) *	52.1 ± 10.3 (24–74)	50.58 ± 11.37 (25–72)
Gender (M/F) *	47/53	30/22
NSAID ingestion *	21 (21.0%)	12 (23.1%)
Smoking *	17 (17.0%)	10 (19.2%)
Alcohol consumption *	7 (7.0%)	6 (11.5%)
Ingestion of tea *	30 (30.0%)	19 (36.5%)
Ingestion of coffee *	39 (39.0%)	20 (38.5%)
BMI (kg/m^2^, mean ± SD/range) *	25.9 ± 4.4 (17.5–36.5)	25.7 ± 4.8 (17.8–38.5)
Peptic ulcer disease *	28 (28.0%)	15 (28.8%)
**Gastric Juice pH ≥ 6.0**	45 (45.0%) **	51 (98.1%) **
**Eradication rate**	51/95 (53.7%) ***	44/52 (84.6%) ***

ILTHPI: intraluminal therapy for *Helicobacter pylori* infection using medicaments containing amoxicillin, metronidazole, and clarithromycin. SD: standard deviation. NSAID: non-steroid anti-inflammatory drug. BMI: Body Mass Index. ^†^ Group A: ILTHPI without acid control. ^†^ Group B: ILTHPI with acid control. * *p* > 0.05 for all clinical characteristics between Group A and Group B. ** *p* < 0.0001 for the percentage of gastric juice pH ≥ 6.0 comparing Group A versus Group B. *** *p* = 0.0004 for the eradication rate comparing Group A versus Group B.

**Table 6:** Comparison of medicament containing Tetracycline, Metronidazole, and Bismuth versus Amoxicillin, Metronidazole, and Clarithromycin for ILTHPI.

Medicament Containing	Stomach Acid Control before ILTHPI (Eradication Rate)	Adverse Events of ILTHPI	Oral Antibiotic Therapy for ILTHPI Failure (Eradication Rate)	ILTHPI Plus Oral Antibiotic Therapy for ILTHPI Failure (Overall Eradication Rate)
**Tetracycline 2 g** **Metronidazole 2 g** **Bismuth 480 mg**	**Yes** (76.5%; 39/51) *	**1/52 (** **1.9%** **)** *	**7-day concomitant therapy** **(** **83.3%** ; 10/12) *	**49/51 (** **96.1%** **)** *
**Amoxicillin 3 g** **Metronidazole 2 g** **Clarithromycin 1 g**	**Yes** (84.6%; 44/52) *^†^	**2/52** (3.8%) *	**7-day bismuth quadruple** **therapy** ** (87.5%** ; 7/8) *	**51/52 (** **98.1%** **)** *
**Amoxicillin 3 g** **Metronidazole 2 g** **Clarithromycin 1 g**	**No (53.7%** **; 51/95)** **^†^**			
***p*** **value**	* *p* = 0.427 **^†^** ***p* = 0.0004**	* *p* = 1.0	* *p* = 1.0	* *p* = 0.618

ILTHPI: Intraluminal therapy for *Helicobacter pylori* infection. * p (green): Patients with stomach acid control before ILTHPI. † *p* (blue): Comparison of patients with and without stomach acid control before ILTHPI using medicament containing Amoxicillin, Metronidazole, and Clarithromycin.

## Data Availability

Not applicable.
